# Passivation of Sodium Benzenesulfonate at the Buried Interface of a High-Performance Wide-Bandgap Perovskite Solar Cell

**DOI:** 10.3390/ma17071532

**Published:** 2024-03-27

**Authors:** Sijia La, Yaqi Mo, Xing Li, Xuzheng Feng, Xianggang Chen, Zhuoxin Li, Miao Yang, Dongxu Ren, Shuyi Liu, Xiaoxia Cui, Jieqiong Chen, Zhao Zhang, Zhengbo Yuan, Molang Cai

**Affiliations:** 1 State Key Laboratory of Alternate Electrical Power System with Renewable Energy Sources, North China Electric Power University, Beijing 102206, China; 120212211102@ncepu.edu.cn (S.L.); langyue438@163.com (Y.M.); 120222111017@ncepu.edu.cn (X.F.); xianggangchena@163.com (X.C.); lzx51066@163.com (Z.L.); 18101365179@163.com (M.Y.); donxuren@163.com (D.R.); 120222211091@ncepu.edu.cn (S.L.); cxx529925@163.com (X.C.); 120212211009@ncepu.com (J.C.); 120212211013@ncepu.edu.cn (Z.Z.); 120212211051@ncepu.edu.cn (Z.Y.); 2 Institute of Microelectronics, Chinese Academy of Sciences, Beijing 100029, China; lixing2021@ime.ac.cn

**Keywords:** buried interface, inverted wide-bandgap perovskite solar cells, interface passivation

## Abstract

The phase segregation of wide-bandgap perovskite is detrimental to a device’s performance. We find that Sodium Benzenesulfonate (SBS) can improve the interface passivation of PTAA, thus addressing the poor wettability issue of poly[bis(4-phenyl)(2,4,6-trimethylphenyl)amine](PTAA). This improvement helps mitigate interface defects caused by poor contact between the perovskite and PTAA, reducing non-radiative recombination. Additionally, enhanced interface contact improves the crystallinity of the perovskite, leading to higher-quality perovskite films. By synergistically controlling the crystallization and trap passivation to reduce the phase segregation, SBS-modified perovskite solar cells (PSCs) achieved a power conversion efficiency (PCE) of 20.27%, with an open-circuit voltage (*V*_oc_) of 1.18 V, short-circuit current density (*J*_sc_) of 20.93 mA cm^−2^, and fill factor (FF) of 82.31%.

## 1. Introduction

Metal halide perovskite materials exhibit excellent optoelectronic properties and have promising applications in the field of optoelectronics. While single-junction perovskite solar cells (PSCs) have seen unprecedented development, further improvements become challenging as their efficiency approaches the Shockley–Queisser limit [1]. Theoretical calculations have shown that incorporating multiple absorber layers with different bandgaps in a multijunction stacked structure could more effectively utilize the solar spectrum and potentially overcome the efficiency limitations of single-junction solar cells [2]. Currently, the highest efficiency that single-junction cells with wide bandgaps have reached is 22.28% [3]. For highly efficient tandem solar cells, the highest reached efficiency of stacked structures combining wide-bandgap perovskites with silicon is 33.9%. Therefore, wide-bandgap perovskite cells are an ideal choice for preparing stacked cells, offering the potential to surpass the Shockley–Queisser limit. Perovskite solar cells can be classified into n-i-p (normal) and p-i-n (inverted) structures. The traditional formal perovskite solar cells are composed of a conductive glass/electron transport layer/calcium titanium oxide light-absorbing layer/hole transport layer/metal electrode. In contrast, inverted perovskite solar cells are composed of a conductive glass/hole transport layer/calcium titanium oxide light-absorbing layer/electron transport layer/metal electrode. Inverted structures (p-i-n) exhibit superior performance in stacked cells [4], with the highest efficiency reached by single-junction inverted cells being 26.14%, thus receiving increasing attention and being used for an increasing number of applications [5].

Currently, the main limitation to the efficiency of inverted perovskite solar cells lies in the interface energy losses between the perovskite layer and the charge transport layers. Using prevalent solution-based fabrication methods, it is easy to develop defects at the buried interface during the perovskite film’s growth, leading to decreased interface uniformity and non-radiative recombination [6,7]. Excessive interface energy losses can result in a reduced open-circuit voltage of the device [8]. Therefore, reducing defects between the perovskite and the hole transport layers is an effective approach to improving the performance of p-i-n structured devices in inverted perovskite solar cells [9,10,11,12].

Many researchers have improved the wettability of the hole transport layer through interface passivation to reduce defects caused by poor contact between the perovskite layer and the hole transport layer, thereby enhancing a device’s performance and stability. You et al. deposited a discontinuous layer of Al_2_O_3_ on PTAA as a physical surface modifier, improving the wettability of the perovskite on PTAA and achieving a PCE of 17.46% for inverted devices [13]. Poly[(9,9-bis(30-(N,N-dimethylamino)propyl)-2,7-fluorene)-alt-2,7-(9,9-dioctylfluorene)] (PFN) can enhance the coverage of perovskite on poly[N,N′-bis(4-butylphenyl)-N,N′-bis(phenyl)-benzidine] (Poly-TPD) and has been found to be effective on PTAA [14,15]. Poly(methyl methacrylate) (PMMA) passivation interlayers have also been used to improve the wetting of perovskite on PTAA/PMMA, achieving efficiencies of over 19% using pristine PTAA [16]. Zhang et al. spin-coated PbI_2_ onto PTAA, and the PbI_2_ that was grafted onto the PTAA film preferentially interacted with MAI in the perovskite precursor solution and formed a perovskite structure by sharing iodine atoms. The attraction between PbI_2_ and MAI could compensate for the surface tension of the precursor solution on the PTAA film, resulting in a hydrophilic PTAA film surface that allowed the perovskite precursor solution to spread freely on the PTAA [17].

In this study, the organic polar material Sodium Benzenesulfonate (SBS) is introduced at the interface between the PTAA layer and the perovskite layer. SBS contains sulfonate functional groups that passivate defects and lack halide ions [18], thereby avoiding an exacerbation of the halide unevenness at the bottom interface. After SBS passivation, the wettability of the PTAA layer is improved, perovskite crystallization is enhanced, and the film quality is increased. Moreover, due to the passivation effect of SBS sulfonate functional groups, non-radiative recombination in the perovskite film is reduced. After the interaction of SBS with PTAA, the hole extraction capability of the modified PTAA layer is enhanced, leading to an increase in the power conversion efficiency (PCE) of the devices from 18.99% to 20.27% after bottom interface modification.

## 2. Materials and Methods

Materials: Poly[bis(4-phenyl) (2,4,6-trimethylphenyl) amine] (PTAA), fullerene (C60), and 2,3,5,6-Tetrafluoro-7,7′,8,8′-tetracyanoquinodimethane (F4-TCNQ) were purchased from Xi’an Baolaite Optoelectronics Technology Co., Ltd. (Xi’an, China). N,N-Dimethylformamide (DMF), anisole (CH_3_OC_6_H_5_), chlorobenzene, and dimethyl sulfoxide (DMSO) were acquired from Energy Chemical (Chuzhou, China). Lead iodide (PbI_2_) was obtained from 3A Company (Sins, Switzerland). Formamidinium iodide (FAI) was sourced from Xi’an Huada Zhiyuan Technology Co., Ltd. (Xi’an, China), methylammonium bromide (MABr) from Greatcell Solar Company (Queanbeyan, Australia), lead bromide (PbBr_2_) from Macklin (Shanghai, China), cesium iodide (CsI) from Aladdin (Shanghai, China), Sodium Benzenesulfonate (SBS) from Alfa Aesar (Haverhill, MA, USA), and bathocuproine (BCP) from Sigma-Aldrich (St. Louis, MO, USA).

Indium tin oxide (ITO) glass was cleaned sequentially with detergent, deionized water, acetone, and anhydrous ethanol. After drying, the ITO was treated with ultraviolet ozone for 15 min. Subsequently, PTAA was spin-coated at 5000 rpm for 30 s and annealed on a hotplate in a nitrogen atmosphere at 100 °C for 10 min. Here, a 1.3 M perovskite precursor solution was prepared by mixing CsI (0.065 mM), FAI (0.8645 mM), MABr (0.3705 mM), PbI_2_ (1.0953 mM), and PbBr_2_ (0.2048 mM) in 1 mL of DMF/DMSO (4:1 volume ratio) mixture and stirred for 5 h in a nitrogen atmosphere. SBS (5, 10, 15 mM) was dissolved in DMF. Before spin-coating the perovskite precursor solution, solutions of SBS at different concentrations were spin-coated (4000 rpm, 30 s) onto the annealed PTAA substrate, followed by immediate spin-coating (1000 rpm, 5 s/4000 rpm, 30 s) to deposit the perovskite film, using 100 μL of anisole as an anti-solvent 15 s before the end of the spin-coating process. The device was prepared by sequentially depositing C60 (30 nm), BCP (5 nm), and a silver electrode (80 nm) through thermal evaporation under high-vacuum conditions.

## 3. Results and Discussion

**Figure 1:**
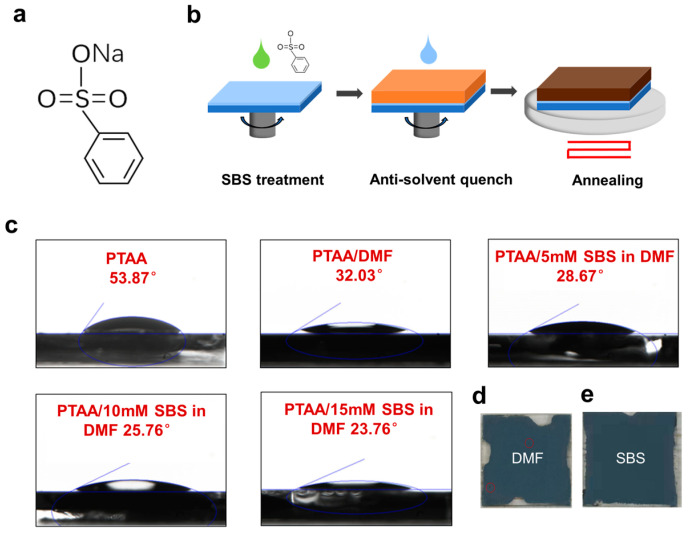
(**a**) Chemical structure diagram of SBS. (**b**) Schematic diagram of process for SBS-modified PTAA/perovskite interface. (**c**) Contact angle measurement graph of PTAA without pretreatment and treated with DMF, 5 mM SBS solution, 10 mM SBS solution, and 15 mM SBS solution. (**d**) Photograph of perovskite film prepared on PTAA substrate treated with DMF. (**e**) Photograph of perovskite film prepared on PTAA substrate treated with SBS solution.

The molecular formula of Sodium Benzenesulfonate is shown in Figure 1a, and the schematic diagram of the modification process is illustrated in Figure 1b. In conventional processes, DMF is spin-coated onto the PTAA substrate before spinning the perovskite thin film to enhance its wettability. In this study, SBS is dissolved in a DMF solvent for the pretreatment of the PTAA substrate. The samples treated with DMF are referred to as Control samples in the following description. The samples treated with SBS solution are referred to as SBS samples. As shown in Figure 1c, the wettability of different PTAA film surfaces was characterized by measuring the contact angle of the perovskite droplets on PTAA substrates that were treated with various methods. The contact angle of the perovskite droplets on the PTAA spin-coated with DMF is as high as 53.87°, indicating that it is challenging for the precursor solution to spread uniformly on the substrate surface. After the PTAA films were treated with DMF, the contact angle decreased to 32.03°. However, the coverage of the perovskite films at the edge positions remained poor, and many holes appeared (highlighted by red circles in the figure), indicating that the PTAA still exhibited strong hydrophobicity after the DMF treatment, which is unfavorable for the fabrication of efficient perovskite solar cells (Figure 1d). The enlarged view of Figure 1d is shown in Figure S1. After treating PTAA with different concentrations of SBS, the contact angle decreased from 28.67° to 23.76° with an increasing SBS concentration. The contact angle was smaller than that of the untreated PTAA films and PTAA films treated with DMF, indicating that the perovskite films that were passivated by SBS exhibited more complete and bright coverage, with a reduced number of holes, resulting in nearly fully covered and uniform black perovskite films (Figure 1e). This suggests that SBS modification can improve the wettability of PTAA to obtain high-quality and highly covered perovskite films. This also enables perovskite to evenly cover PTAA with good contact while reducing recombination centers and carrier trap structure defects.

**Figure 2:**
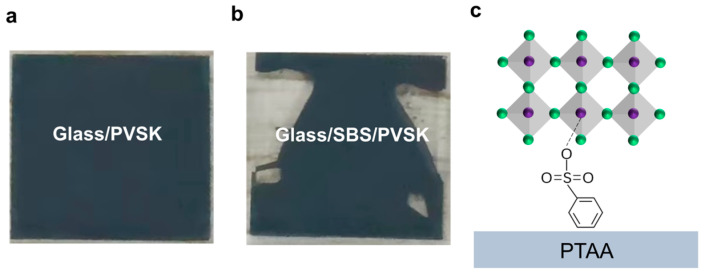
(**a**) Photograph of glass/DMF/perovskite film. (**b**) Photograph of glass/SBS/perovskite film. (**c**) Schematic diagram of SBS serving as chemical bridge between PTAA substrate and perovskite layer.

To further investigate the mechanism by which SBS improves a film’s quality and enhances its interface, quartz glass was used as the substrate instead of PTAA to prepare the perovskite films. The perovskite films were fabricated on quartz glass substrates treated with UV/ozone. Samples treated with DMF achieved complete coverage of the perovskite films, while those treated with the SBS solution showed numerous void areas (Figure 2a,b), in contrast to the phenomenon observed with PTAA substrates. The DMF-treated glass exhibited good wettability, while the SBS-treated glass showed increased hydrophobicity, possibly due to the hydrophobic nature of the benzene ring group in SBS. The SBS layer may prevent the perovskite solution from adhering during the spinning process. This phenomenon suggests an interaction between the SBS and PTAA. Based on the above characterization, a reasonable speculation can be made regarding the mechanism of the SBS-modified bottom interface: the sulfonate end of SBS anchors the perovskite through a S=O-Pb coordination bond, while the benzene ring group on the other end interacts with PTAA (Figure 2c). SBS establishes a chemical bridge at the PTAA/perovskite interface, passivating uncoordinated Pb2+ ions while enhancing hole transport.

**Figure 3:**
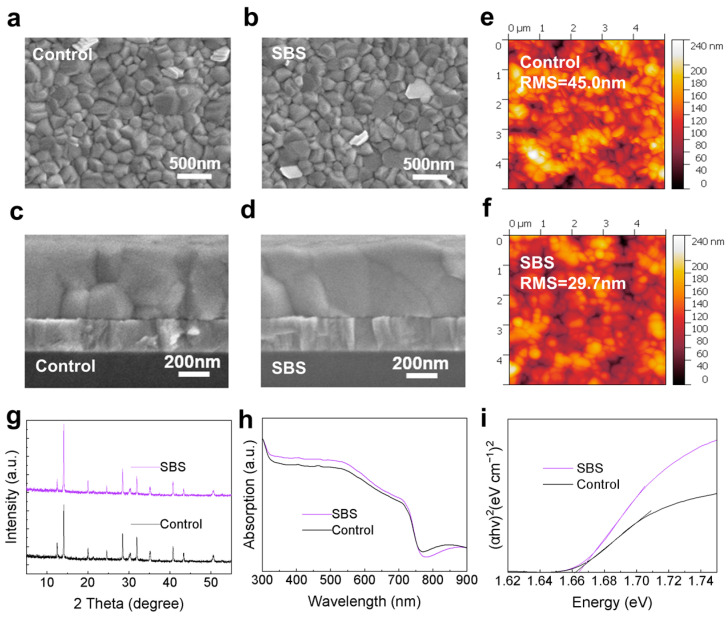
(**a**) SEM image of Control film’s and (**b**) SBS-passivated perovskite film’s surface. (**c**) SEM image of Control film’s cross-section and (**d**) SBS-passivated perovskite film’s cross-section. (**e**) AFM image of Control film and (**f**) SBS-passivated perovskite film. (**g**) XRD diffraction patterns of Control and SBS-passivated perovskite films. (**h**) UV–visible absorption spectra. (**i**) Tauc plot spectra.

To investigate the effect of SBS modification on the morphology of the perovskite, SEM characterization was conducted on the surface and cross-section of the Control perovskite films and the SBS-passivated perovskite films. As shown in Figure 3a,b, compared to the Control film, the grain size and surface morphology of the SBS-passivated film did not show significant changes. However, as observed in Figure 3c,d, the grains in the SBS-passivated film appeared more regular and well-connected, with enhanced interface bonding. To further verify that the grain size of the perovskite film did not change significantly after SBS passivation, we conducted statistical analysis of the grain size distribution based on the SEM images in Figure 3a,b, as shown in Figure S2. As depicted in Figure S2a,b, the majority of grain sizes in both the Control and SBS-treated films were in the range of 200–300 nm. However, compared to the Control film, the distribution of grain sizes in the SBS-treated film shows a slightly higher proportion in the 300 nm range, while the Control film shows a slightly higher proportion around 250 nm. Therefore, we conclude that SBS passivation results in a slight increase in the grain size of the perovskite film. The main peak at 13.9° in the XRD spectra (Figure 3g) also showed a noticeable enhancement, indicating an improvement in the perovskite crystallinity after SBS modification. Table S1 lists the intensities of the three strongest characteristic peaks. A comparison of these values reveals that the crystal orientation at 100 and 200 is enhanced after SBS passivation, indicating an improvement in the crystals’ crystallinity. The modification with SBS contributes to the enhancement of the perovskite film’s quality. In order to further validate the enhancement of crystallinity after SBS passivation, we conducted a Williamson–Hall (WH) analysis of the powder scraped from the perovskite film that was deposited on the SBS-passivated bottom interface. As shown in Figure S4, the residual lattice strain can be determined from the slope of the fitting line, which decreased from 0.00213 to 0.00159 in the films without and with SBS passivation, respectively. The SBS passivation at the bottom interface improves the wettability with the improved PTAA interface, leading to better growth of the perovskite film. Consequently, lattice distortion is eliminated, releasing the lattice strain within the perovskite’s bottom film. We believe that the sulfonic acid group at the end of the SBS molecule anchors the perovskite via S=O-Pb coordination bonds, which preferentially interact with Pb in the perovskite precursor solution. SBS anchored on the PTAA film may serve as nucleation sites for perovskite crystal growth, thereby promoting the enhancement of perovskite crystallinity. The strengthened interface bonding facilitates hole transport at the interface, as will be discussed later. The improvement in wettability of the PTAA substrate leads to a smoother and more uniform perovskite film coverage. Consequently, the roughness of the perovskite film decreased from 37.60 nm to 33.80 nm, resulting in an enhancement of the film’s quality (Figure 3e,f). To further validate the improved grain quality of the film after SBS passivation, we processed the 2D AFM images of Figure 3e,f into 3D representations, as shown in Figure S3. We found that the surface roughness of the Control film reached 0.24 μm (Figure S3a), while the surface roughness of the SBS film was only 0.21 μm, with more regular grains (Figure S3b). This indicates that the surface of the Control film was rough, while the surface of the SBS film was more uniform, resulting in a better film quality. As shown in Figure 3h, the UV-Vis absorption spectra indicate an increase in absorbance for the SBS-passivated film, corresponding to the enhanced crystallinity of the perovskite film. Utilizing the Tauc plot method to extrapolate (Ahv)^2^ vs. hv from the absorption spectra of the Control and SBS samples (Figure 3i), the bandgaps of both films were found to be consistent [19,20,21], indicating that SBS modification of the bottom interface enhances light absorption without affecting the band structure of the perovskite layer. Overall, these results suggest that pretreatment of the substrate with SBS can improve the quality of the perovskite film.

The average recombination lifetime (*τ*_ave_) is estimated from the fitting data using a bi-exponential decay function, as described by the following equation [22]:

(1) $$\tau_{ave}=\frac{{\sum A_{i}\tau_{i}}^{2}}{\sum A_{i}\tau_{i}}$$

where *τ*_i_ is the decay time and *A*_i_ is the amplitude.

**Figure 4:**
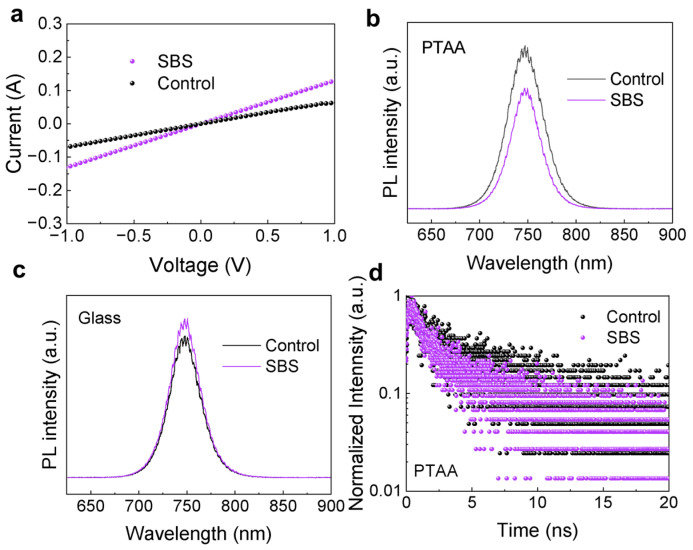
(**a**) The dark J-V curves of the ITO/PTAA/Ag device and the ITO/PTAA/SBS/Ag device. (**b**) The PL spectra of the perovskite film on PTAA treated with DMF and SBS solutions. (**c**) The PL spectra of the perovskite film on quartz glass treated with DMF and SBS solutions. (**d**) The TRPL decay curves of the perovskite film treated with DMF and SBS solutions.

The electrical conductivity of both the PTAA and the PTAA after SBS passivation was tested. The formula for calculating electrical conductivity is as follows:

(2) $$\sigma =\frac{Id}{VA}$$

where *A* (0.04 cm^2^) is the area of the sample and *d* is the thickness of the sample. The relevant calculation parameters are shown in Table 1. As depicted in Figure 4a, the calculated electrical conductivity of PTAA/SBS is 3.25 × 10^−4^ mS cm^−1^, and the electrical conductivity of PTAA is 1.65 × 10^−4^ mS cm^−1^. The increase in enhanced electrical conductivity demonstrates that PTAA/SBS has a stronger charge transport capability, effectively inhibiting charge accumulation at the PTAA/PVSK interface.

Pure hole-only devices with PTAA substrates and SBS-treated perovskite films were prepared for PL testing. As shown in Figure 4b, the SBS film exhibited stronger fluorescence quenching, indicating that the photoexcited holes in the SBS film can more effectively transfer from the perovskite to the PTAA substrate. Figure 4c shows the photoluminescence (PL) spectra of the perovskite films, which were deposited on glass substrates treated with DMF and SBS solutions, respectively. The PL intensity of the films based on SBS modification is higher than that of the Control films. The increased PL intensity of the films after SBS modification of the PTAA indicates a reduction in non-radiative recombination. This can be attributed to the improved crystalline quality of the perovskite due to SBS modification, and the sulfonic acid groups in SBS can provide lone pairs of electrons to form coordinate bonds with Pb^2+^, thus passivating defects.

To further validate this conclusion, time-resolved photoluminescence (TRPL) measurements were conducted (Figure 4d). The corresponding fitting parameters are shown in Table 2. The decay of *τ*_1_ is mainly determined by the quenching of carriers at the interface, while *τ*_2_ is mainly determined by the radiative recombination of carriers that is caused by defects in the perovskite [23]. The results showed that *τ*_1_ decreased from 0.72 ns to 0.23 ns after SBS passivation, indicating that SBS passivation improved the interface contact, thereby leading to faster carrier quenching at the interface. Additionally, *τ*_2_ decreased from 5.66 ns to 1.12 ns, suggesting that SBS passivation improved the quality of the perovskite film, resulting in a reduction in internal defects within the perovskite. The results show that after introducing SBS at the bottom interface, both *τ*_1_ and *τ*_2_ decrease, and the average decay lifetime decreases from 2.24 ns to 0.45 ns, indicating faster interface charge transfer due to improved interface contact in SBS samples.

**Figure 5:**
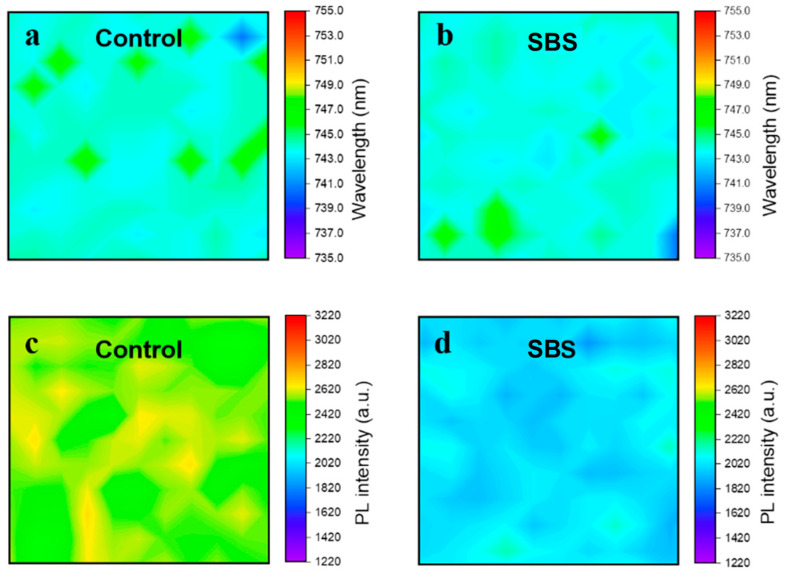
(**a**) Micro-area photoluminescence (PL) spectra scans of perovskite films of Control and (**b**) SBS-modified perovskite films. (**c**) Micro-area photoluminescence (PL) intensity scans of perovskite films of Control and (**d**) SBS-modified perovskite films.

Utilizing micro-area photoluminescence (PL) spectra scans of the perovskite films, the effect of SBS on the passivation of the substrate was observed in the planar region. Compared to the SBS-modified samples, the Control films exhibit more red-shifted regions in the PL peak positions, possibly due to a lower degree of phase segregation resulting from a poorer film quality (Figure 5a,b). Figure 5c,d display the corresponding fluorescence intensity scans, indicating a decrease in PL intensity for the SBS-modified films, demonstrating that SBS can uniformly adhere to the buried interface, rather than just locally passivating it.

**Figure 6:**
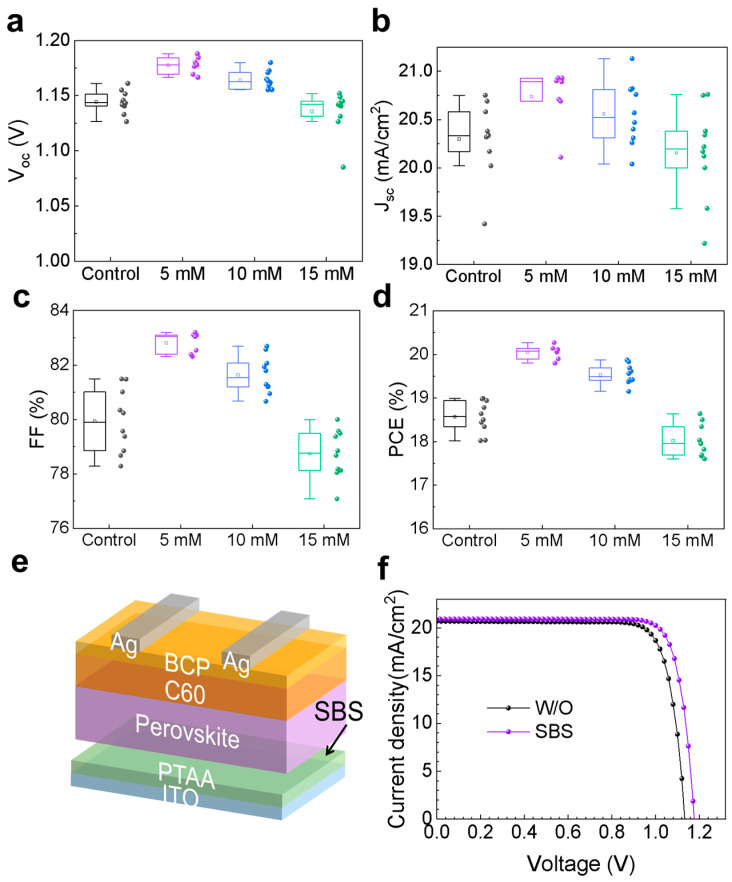
The photovoltaic parameters of the perovskite solar cells based on SBS bottom interface modification, including the (**a**) open-circuit voltage (*V*_oc_), (**b**) short-circuit current density (*J*_sc_), (**c**) fill factor (*FF*), (**d**) power conversion efficiency (PCE), and (**e**) schematic diagram of the device structure with SBS bottom interface modification. (**f**) The J-V curves of the devices for both Control and SBS-treated samples.

To understand the influence of SBS bottom interface modification on the photovoltaic performance of the devices, we fabricated p-i-n-structured PSCs using SBS solutions of different concentrations (5, 10, and 15 mM). Figure 6a–d show the statistical photovoltaic parameters of the perovskite solar cells based on SBS bottom interface modification. The *V*_oc_ of the devices increases when they are based on 5 mM and 10 mM SBS modification, while it decreases when the concentration is increased to 15 mM. The device with 5 mM SBS modification exhibits good repeatability in *J*_sc_, and the most significant improvement in PCE is observed. The J-V curves of the devices are shown in Figure 6f, with relevant performance parameters summarized in Table 3. Upon introducing SBS at the bottom interface, the *V*_oc_ and *FF* of the perovskite devices increase from 1.14 V and 81.01% to 1.18 V and 82.31%, respectively, while the PCE increases from 18.99% to 20.27%. The perovskite devices that were modified with SBS exhibit better photovoltaic performance, attributed to the improved crystal quality of the perovskite, lower charge carrier losses, and tighter interface bonding.

**Figure 7:**
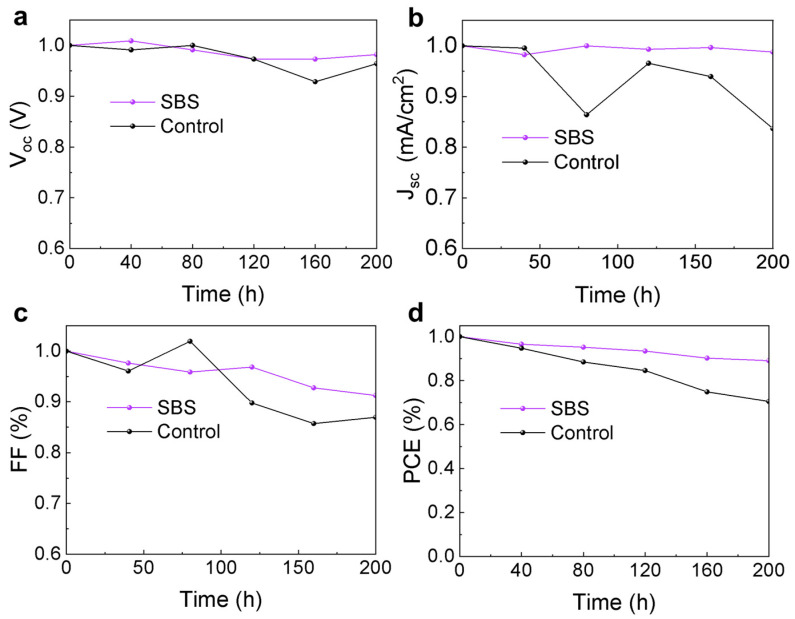
The variation in (**a**) *V*_oc_, (**b**) *J*_sc_, (**c**) *FF*, and (**d**) PCE over time for the Control and SBS devices under AM1.5 illumination (ambient temperature 15–20 °C, humidity 15–20% RH).

This study evaluated the effect of SBS interface passivation on the stability of devices. Encapsulated perovskite solar cells were exposed to a xenon lamp light source with an intensity of 100 mW/cm^2^ at an ambient temperature of 15–20 °C and relative humidity of 10–15%. The variation in each photovoltaic parameter over time is shown in Figure 7. After 200 h of light exposure, the efficiency of the Control device decreased to 70.5% of its initial efficiency, while the efficiency of the SBS device was 89.1% of its initial efficiency after aging. The improvement in device stability after SBS modification can be attributed to the enhancement of the perovskite film’s quality and the interface binding by SBS.

## 4. Conclusions

We found that SBS passivation at the interface with PTAA can improve the poor wettability of PTAA, thereby addressing the interface defect issues caused by poor contact between the perovskite and PTAA and reducing non-radiative recombination. Moreover, the improved interface contact can enhance the crystallinity of the perovskite, leading to an improvement in the quality of the perovskite film. By controlling the crystallization and trap passivation, a synergistic effect is achieved in enhancing the performance of PSCs. Moreover, due to the passivation effect of the SBS sulfonate functional groups, the non-radiative recombination in the perovskite film is reduced. After the interaction of SBS with PTAA, the hole extraction capability of the modified PTAA layer is enhanced. As a result, PSCs that are modified with SBS achieved a PCE of 20.27%, along with a *V*_oc_ of 1.18 V, a *J*_sc_ of 20.93 mA/cm^2^, and a *FF* of 82.31%.

## Figures and Tables

**Table 1:** Conductivity of ITO/PTAA/Ag devices and ITO/PTAA/SBS/Ag devices.

HTL	Slope (mS cm^−2^)	Thickness (nm)	Conductivity (mS cm^−1^)
Control	165.00	10	1.65 × 10^−4^
SBS	325.00	10	3.25 × 10^−4^

**Table 2:** TRPL decay lifetimes of perovskite films deposited on PTAA and PTAA/SBS substrates.

Samples	*τ*_1_ (ns)	*A*_1_	*τ*_2_ (ns)	*A*_2_	*τ*_ave_ (ns)	Chi-Squared
Control	0.72	69.2%	5.66	30.8%	2.24	0.0038
SBS	0.23	75.5%	1.12	24.5%	0.45	0.0017

**Table 3:** Photovoltaic parameters of PTAA devices with different concentrations of SBS modification and Control devices.

	*V*_oc_ (V)	*J*_sc_ (mA/cm^2^)	*FF* (%)	PCE (%)
Control	1.14	20.69	81.01	18.99
5 mM	1.18	20.93	82.31	20.27
10 mM	1.17	20.82	81.93	19.85
15 mM	1.14	20.75	78.68	18.64

## Data Availability

The data that support the findings of this study are available from the corresponding author upon reasonable request.

## Supplementary Materials

The following supporting information can be downloaded at: https://www.mdpi.com/article/10.3390/ma17071532/s1, Figure S1: Enlarged photograph of the perovskite film prepared on a PTAA substrate treated with SBS solution. Figure S2: The grain sizes of the perovskite films on (a) PTAA and (b) PTAA substrates treated with SBS. Figure S3: 3D AFM images of (a) Control film and (b) SBS-passivated perovskite film. Figure S4: The Williamson-Hall (WH) plots of the powders scraped from the untreated and SBS-passivated perovskite films. Table S1: The diffraction peak indices of the Control and SBS-treated perovskite films.
