# Supplementary material for: Passivation of Sodium Benzenesulfonate at the Buried Interface of a High-Performance Wide-Bandgap Perovskite Solar Cell
Source: Materials (Basel). 2024 Mar 27;17(7):1532. doi: 10.3390/ma17071532 (PMC11012805; doi:10.3390/ma17071532)
Supplement: Supplementary file 1 [file materials-17-01532-s001.zip › materials-2926961-supplementary.pdf]

## Supplementary Results

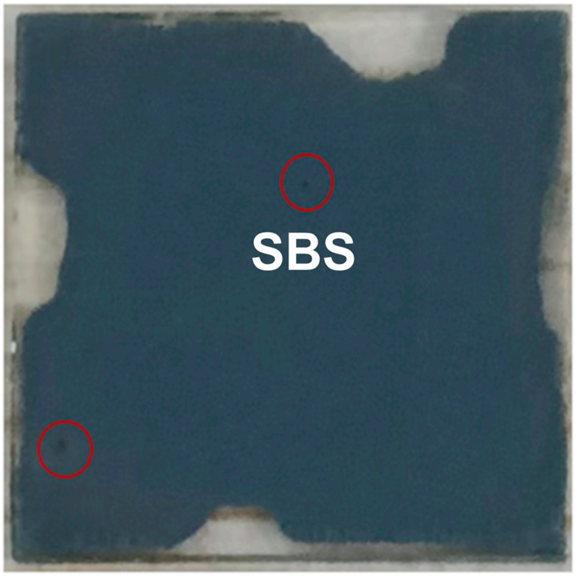


Figure S1. Enlarged photograph of the perovskite film prepared on a PTAA substrate treated with SBS solution.

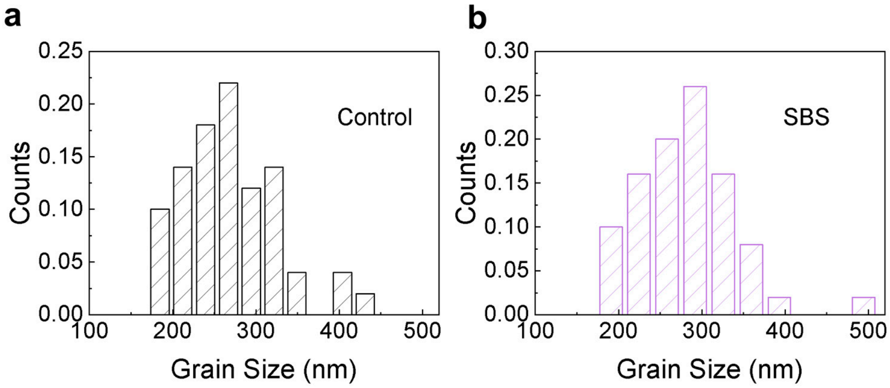


Figure S2. The grain sizes of the perovskite films on a) PTAA and b) PTAA substrates treated with SBS.

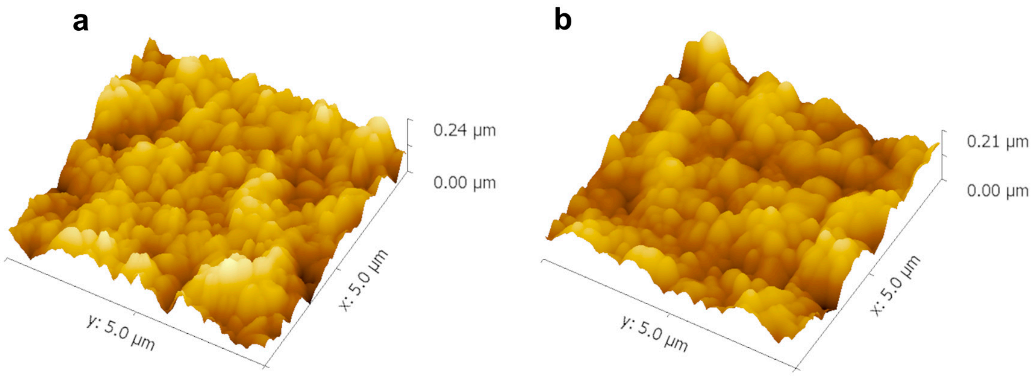


Figure S3. 3D AFM images of a) Control film and b) SBS-passivated perovskite film.

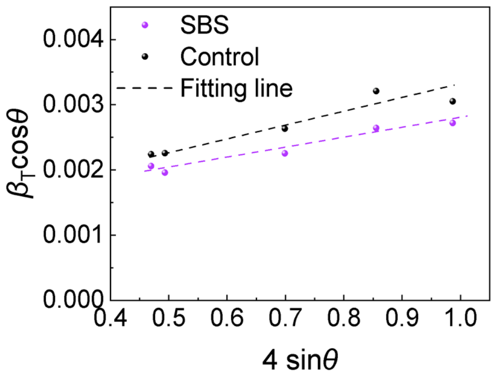


Figure S4. The Williamson-Hall (WH) plots of the powders scraped from the untreated and SBS-passivated perovskite films.

TableS1. The diffraction peak indices of the Control and SBS-treated perovskite films.

| The crystal planes | 2θ (°) | Peak intensity | |
|---|---|---|---|
| | | Control | SBS |
| (100) | 14.0 | 23634.1 | 33087.7 |
| (200) | 28.5 | 19430.9 | 20264.2 |
| (210) | 31.8 | 16128.3 | 14730.9 |
